# Hsp104 Suppresses Polyglutamine-Induced Degeneration Post Onset in a Drosophila MJD/SCA3 Model

**DOI:** 10.1371/journal.pgen.1003781

**Published:** 2013-09-05

**Authors:** Mimi Cushman-Nick, Nancy M. Bonini, James Shorter

**Affiliations:** 1 Department of Biology, University of Pennsylvania, Philadelphia, Pennsylvania, United States of America; 2 Department of Biochemistry and Biophysics, Perelman School of Medicine, University of Pennsylvania, Philadelphia, Pennsylvania, United States of America; 3 Neuroscience Graduate Group, Perelman School of Medicine at the University of Pennsylvania, Philadelphia, Pennsylvania, United States of America; 4 Howard Hughes Medical Institute, University of Pennsylvania, Philadelphia, Pennsylvania, United States of America; Stanford University School of Medicine, United States of America

## Abstract

There are no effective therapeutics that antagonize or reverse the protein-misfolding events underpinning polyglutamine (PolyQ) disorders, including Spinocerebellar Ataxia Type-3 (SCA3). Here, we augment the proteostasis network of *Drosophila* SCA3 models with Hsp104, a powerful protein disaggregase from yeast, which is bafflingly absent from metazoa. Hsp104 suppressed eye degeneration caused by a C-terminal ataxin-3 (MJD) fragment containing the pathogenic expanded PolyQ tract, but unexpectedly enhanced aggregation and toxicity of full-length pathogenic MJD. Hsp104 suppressed toxicity of MJD variants lacking a portion of the N-terminal deubiquitylase domain and full-length MJD variants unable to engage polyubiquitin, indicating that MJD-ubiquitin interactions hinder protective Hsp104 modalities. Importantly, in staging experiments, Hsp104 suppressed toxicity of a C-terminal MJD fragment when expressed after the onset of PolyQ-induced degeneration, whereas Hsp70 was ineffective. Thus, we establish the first disaggregase or chaperone treatment administered *after* the onset of pathogenic protein-induced degeneration that mitigates disease progression.

## Author Summary

There are no effective therapeutics for any of the neurodegenerative disorders caused by expanded polyglutamine (PolyQ) tracts including Spinocerebellar Ataxia Type-3 (SCA3). These disorders are connected with the misfolding and aggregation of proteins bearing expanded PolyQ tracts in the neurons of affected individuals. In SCA3, ataxin-3 (MJD) is the protein that bears the PolyQ expansion and forms insoluble aggregates. Here, as a therapeutic strategy we introduce Hsp104, a powerful protein disaggregase from yeast, into *Drosophila* models of SCA3. Hsp104 has no homologue in animals, but has an unusual ability to dissolve PolyQ aggregates *in vitro*, an activity that could be harnessed therapeutically. Indeed, Hsp104 suppressed degeneration caused by a C-terminal ataxin-3 (MJD) fragment containing the pathogenic expanded PolyQ tract, which accumulates in disease. However, Hsp104 enhanced aggregation and toxicity of full-length pathogenic MJD. Hsp104 rescued forms of MJD unable to engage polyubiquitin or with a deletion in the deubiquitylase domain indicating that MJD-ubiquitin interactions hinder protective Hsp104 activities. Importantly, Hsp104 suppressed toxicity of a C-terminal MJD fragment when expressed *after* the onset of PolyQ-induced degeneration, whereas Hsp70 was ineffective. Thus, we establish the first disaggregase or chaperone treatment administered *after* the onset of pathogenic protein-induced degeneration that mitigates disease progression.

## Introduction

Many neurodegenerative diseases, such as Alzheimer's Disease, Parkinson's Disease (PD), prion disease, and the collection of polyglutamine (PolyQ) disorders, including Huntington's Disease (HD) and the Spinal Cerebellar Ataxias (SCAs), are characterized by the formation of protein inclusions in the nervous system [1]–[3]. Moreover, despite vastly different primary sequences, many of the proteins implicated in these diseases adopt the stereotypical amyloid conformation in the aggregated state [1]. Amyloid is defined by a highly stable cross-β conformation, in which proteins polymerize via intermolecular contacts of β-strands that align orthogonal to the fiber axis. Amyloid is typically a stable structure that is resistant to denaturation by heat, detergents (up to 2% sodium dodecyl sulfate (SDS)), and proteases [2], [4].

Despite the extraordinary structural stability of amyloid, a protein disaggregase from yeast, Hsp104, can rapidly solubilize amyloid. Hsp104 is a hexameric AAA+ (ATPases Associated with diverse cellular Activities) protein that couples ATP hydrolysis to translocation of substrate through a central pore, thus prying individual monomers from the amyloid fiber [5]–[8]. In yeast, Hsp104 is a heat shock protein (HSP), promoting survival following stresses by resolubilizing denatured protein aggregates and restoring proteins to native form and function [9], [10]. Hsp104 also maintains beneficial prion states by controlling the disassembly and dissemination of amyloid aggregates [11]–[13].

Curiously, Hsp104 has no homologue in metazoa. Indeed, until recently it was unclear whether the metazoan proteostasis network possessed any coupled protein disaggregase and reactivation machinery. It is now clear that Hsp110, Hsp70, and Hsp40 collaborate to promote the dissolution and reactivation of disordered aggregates [14], [15], and can even slowly depolymerize amyloid fibrils from their ends [16]. However, these disaggregase activities are slow and ineffective compared to Hsp104 [15], [16]. In particular, amyloid depolymerization by Hsp110, Hsp70, and Hsp40 is many orders of magnitude slower (weeks versus minutes) than amyloid dissolution by Hsp104 [16]. Importantly, Hsp104 can synergize with metazoan Hsp110, Hsp70, and Hsp40 to promote dissolution of amyloid and nonamyloid aggregates [15], [16]. Thus, introduction of Hsp104 into an animal system may provide an unprecedented opportunity to directly and rapidly target the intractable protein aggregates that underlie amyloid diseases [17], [18].

Spinocerebellar Ataxia Type 3 or Machado-Joseph Disease (MJD/SCA3) is the most prevalent dominantly inherited ataxia [19], [20]. The genetic basis of MJD/SCA3 is an expansion of the polyglutamine (PolyQ) tract of ataxin-3 (also known as Machado-Joseph Disease protein; MJD). When the PolyQ tract surpasses 50 consecutive Qs it is associated with the formation of amyloid aggregates and development of disease [21]–[23]. The normal physiological function of MJD is as a deubiquitylase (DUB) that catalyzes the cleavage of polyubiquitin (poly-ub) chains to promote proteostasis. It has a chain-editing function, preferentially cleaving certain poly-ub linkages to increase the presence of poly-ub chains that signal for degradation via the ubiquitin-proteasome system (UPS) [24], [25]. MJD has DUB activity in the N-terminal Josephin domain, plus two ubiquitin-interacting motifs (UIMs) that present poly-ub chains to the Josephin domain, as well as the C-terminal PolyQ tract that is associated with disease [26]. The PolyQ domain is known to form amyloid fibers, and interestingly, MJD aggregation occurs in a two-step process *in vitro*, with the Josephin domain forming SDS-soluble linear polymers that then convert into SDS-insoluble PolyQ-driven amyloid fibers [27]–[29]. As such, Hsp104 may be well suited to combating MJD protein aggregation because it antagonizes non-amyloid aggregates, pre-amyloid conformers, and amyloid fibers [5], [13], [30], [31].

Hsp104 has been introduced to combat protein-aggregation disease in metazoan systems with various levels of success [31]–[35]. In *C. elegans*, Hsp104 prevented aggregation and toxicity of GFP-tagged PolyQ [33]. In a lentiviral rat model, co-expression of Hsp104 with a PolyQ fragment implicated in HD resulted in the accumulation of more but smaller aggregates and rescue of striatal dysfunction [32]. In mouse, animals transgenic for both an HD fragment and Hsp104 showed limited suppression of PolyQ inclusion formation and a lifespan prolonged by ∼20% [34]. While these studies suggest promise for Hsp104 as a therapeutic against disease-associated protein aggregation, none has provided mechanistic insight into how Hsp104 interacts with amyloidogenic proteins in an animal system. Further, studies to date have looked only at antagonism of aggregation by concomitant co-expression of Hsp104. There has not been an evaluation of the potential of Hsp104 to modulate disease phenotypes *in vivo* after aggregates have already formed and degeneration has begun; a situation likely to mimic an actual therapy. Therefore, we created novel Hsp104 *Drosophila* lines to exploit well-characterized models of disease in combination with powerful genetic tools to temporally control the expression of Hsp104 after disease-associated aggregation and degeneration has begun.

Our studies reveal surprisingly distinct interactions of Hsp104 with the full-length versus a truncated version of the MJD protein. Importantly, we establish that Hsp104 possesses the ability to suppress the progression of degeneration when activated subsequent to onset of expression of the disease protein. These data indicate that protein context is central in Hsp104 interactions, and that Hsp104 displays the ability to halt the progression of pre-established disease *in vivo*.

## Results

### Hsp104 mitigates toxicity of truncated MJD, but enhances toxicity of the full-length MJD

**Figure 1:**
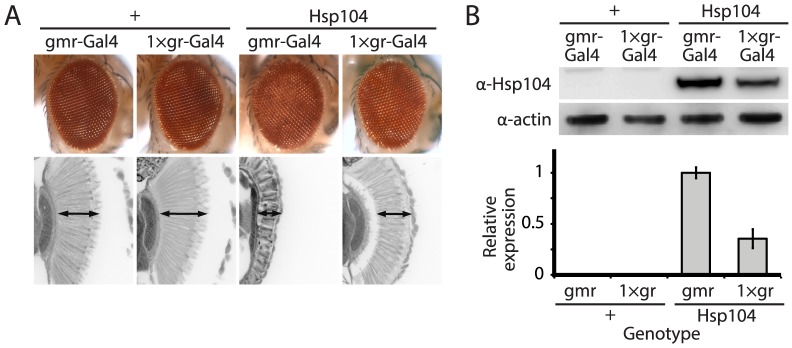
Tuning Hsp104 expression level for the fly eye. (**A**) Expression of UAS-Hsp104 using gmr-GAL4 caused disruption of internal retinal structure by d7. A less strong driver, 1×gr-GAL4 minimized this effect and prevented disruption to cellular organization within the retina. Arrows indicate the width of the retina to highlight changes in tissue integrity. (**B**) Immunoblots demonstrated that 1×gr-GAL4 drives lower Hsp104 expression than gmr-GAL4 at d7. Actin served as a loading control. Quantitation of immunoblots determined that 1×gr-GAL4 levels of Hsp104 were ∼35% that of gmr-GAL4. Hsp104 levels were normalized to actin (n = 3 (mean ± SEM)).

**Figure 2:**
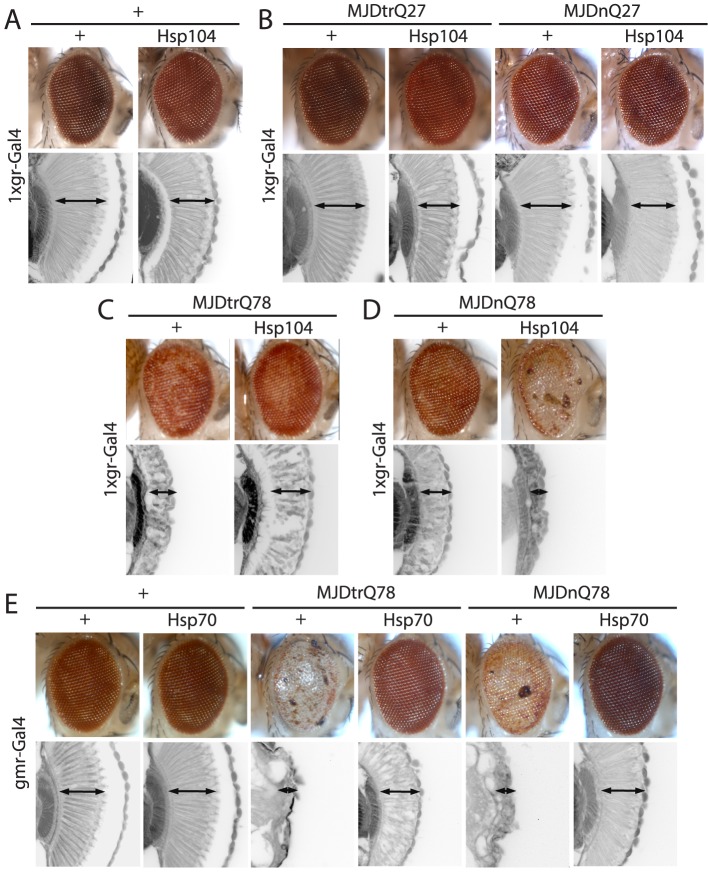
Hsp104 mitigates toxicity of Truncated MJD but enhances toxicity of Full-length MJD. (**A**) With the 1×gr-GAL4 driver at d7, Hsp104 alone had minimal disruption to external eye and internal retinal structure. Arrows indicate the width of the retina to highlight changes in tissue integrity. (**B**) With the 1×gr-GAL4 driver at d7, Hsp104 had no effect on MJD with non-expanded PolyQ tract. Non-pathogenic truncated protein MJDtrQ27 and full-length MJDnQ27 had no toxicity alone, and Hsp104 did not alter this lack of toxicity. The slight disruption to the retina in Hsp104-containing flies is consistent with Hsp104 alone effect. Arrows indicate the width of the retina to highlight changes in tissue integrity. (**C**) With the 1×gr-GAL4 driver at d7, MJDtrQ78 showed moderate toxicity, and co-expression of Hsp104 mitigated the disrupted eye pigmentation and prevented disorganization of internal retinal structure. Arrows indicate the width of the retina to highlight changes in tissue integrity. (**D**) With the 1×gr-GAL4 driver at d7, Hsp104 enhanced toxicity of MJDnQ78, causing loss of pigmentation and dramatic tissue degeneration within the retina. Arrows indicate the width of the retina to highlight changes in tissue integrity. (**E**) With the gmr-GAL4 driver at d7, both MJDtrQ78 and MJDnQ78 displayed severe toxicity, with loss of pigmentation and necrotic patches on the eye (note that severity of degeneration is increased due to the use of the stronger gmr-GAL4 driver). Co-expression of Hsp70 strongly suppressed the toxicity of both full length and truncated pathogenic MJD proteins. Arrows indicate the width of the retina to highlight changes in tissue integrity.

The disaggregase Hsp104 efficiently antagonizes protein aggregates in yeast, and while homologues are present in bacteria, plants, fungi, chromista, and protozoa, no functional homologue has been found in metazoa [17], [36]. We stably introduced Hsp104 into *Drosophila* to evaluate its ability to prevent and potentially reverse aggregation of disease-associated human proteins, readily available in various fly models of disease. To achieve strong expression of the Hsp104 protein in the fruit fly, we codon-optimized the transgene for *Drosophila* (see Materials and Methods), and added a fly-optimal Kozak sequence (ACAAA) before the start codon [37]. The Hsp104 transgene was then expressed in *Drosophila* using the GAL4/UAS system [38]. Because we achieved high expression of Hsp104, expression by the gmr-GAL4 driver in the eye had a mild disruptive effect (Fig. 1A), which has also been observed for another AAA+ protein, p97 [39]. As the gmr-GAL4 driver line has multiple copies of the *glass* gene element for driving GAL4 expression, we instead used a driver line with reduced expression (Fig. 1B) bearing only a single *glass* element, 1×gr-GAL4. Using this driver, the effect of Hsp104 was minimized (Fig. 1A and Fig. 2A). Thus, we used the 1×gr-GAL4 driver line for our experiments to evaluate the impact of Hsp104 on protein-aggregation disease *in vivo*.

Hsp104 dissolves PolyQ amyloid *in vitro* [5], [16] and has been expressed in various PolyQ animal models, with results ranging from minimal beneficial effect to strong abrogation of PolyQ aggregation [32]–[34]. However, a detailed analysis of the underlying protein interactions is lacking *in vivo*. We sought to dissect the ability of Hsp104 to antagonize PolyQ aggregation and toxicity using MJD as a model protein. Pathogenic MJD with expanded PolyQ has been previously established in fly models of MJD/SCA3, and induces progressive neurodegeneration with the formation of nuclear inclusions [40], [41]. We also examined a truncated C-terminal fragment of MJD that is predominantly comprised of the PolyQ tract because fragmentation of the protein may be associated with MJD/SCA3 pathogenesis [40], [42], [43].

We examined interactions of Hsp104 with the pathogenic, full-length MJD containing an expanded glutamine tract (MJDnQ78) and the truncated C-terminal region of the protein containing the expanded glutamine tract (MJDtrQ78) [40]. Hsp104 had no effect on non-pathogenic forms of MJD containing non-expanded PolyQ tracts (Fig. 2B), confirming that the interaction is PolyQ length-dependent. With expanded PolyQ domains, the pathogenic MJDtrQ78 and MJDnQ78 both caused degeneration of the external eye and disruption to internal retinal structure (Fig. 2C, D). Unexpectedly, we found that Hsp104 had opposite effects on these two forms of MJD that have an identical PolyQ expansion: Hsp104 mitigated MJDtrQ78 degeneration (Fig. 2C), yet enhanced degeneration associated with the full-length MJDnQ78 (Fig. 2D). This effect is in contrast to human Hsp70, a molecular chaperone that suppresses PolyQ disease in multiple systems [44]–[47]. Despite more severe degeneration due to stronger expression by the gmr-GAL4 driver, Hsp70 suppressed the toxicity of both MJDtrQ78 and MJDnQ78 (Fig. 2E).

### The opposite effects of Hsp104 on MJDtrQ78 and MJDnQ78 correspond to distinct modulation of underlying protein accumulations

**Figure 3:**
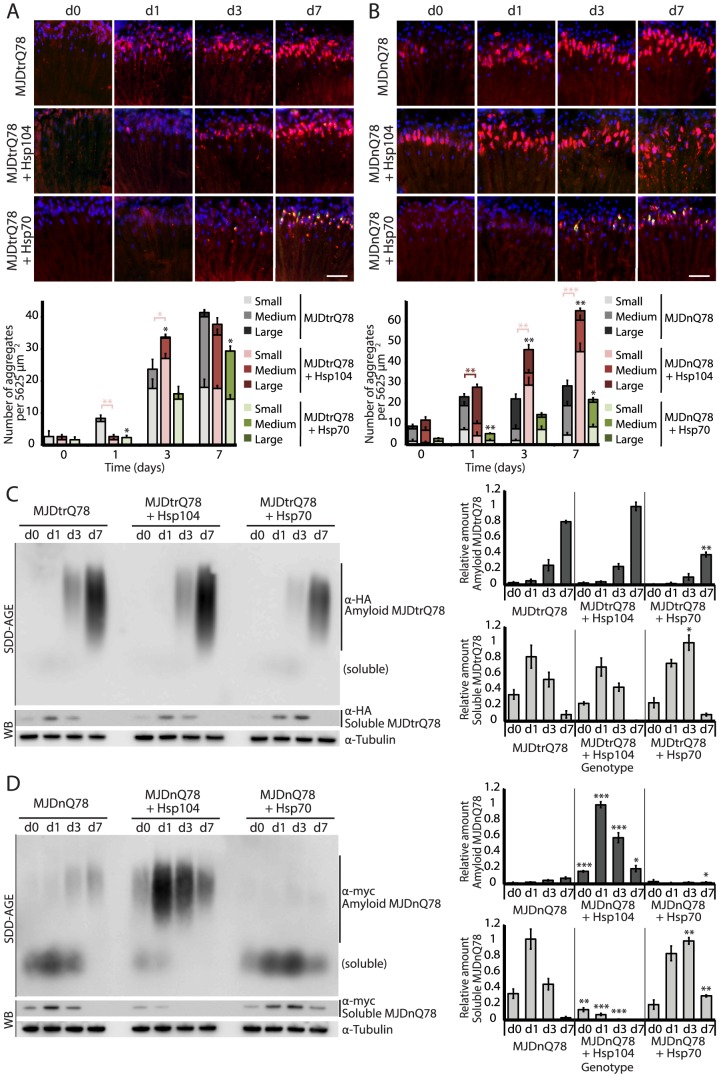
Hsp104 delays aggregation of truncated MJD, but enhances aggregation of full-length MJD. (**A and B**) With the rh1-GAL4 driver at indicated time points, cryosections and IHC demonstrate accumulations of MJDtrQ78 and MJDnQ78 (red) over time, using anti-HA and anti-myc antibodies, respectively. Sections were co-stained with anti-Hsp104 or anti-Hsp70 (green) as indicated, and nuclei were labeled by Hoechst (blue). Hsp104 delayed but did not suppress aggregation of MJDtrQ78, but Hsp104 enhanced accumulation formation of MJDnQ78. Hsp70 suppressed aggregation of both MJD proteins. The size of the aggregates was quantified using ImageJ, with delineations for large inclusions (>5 µm across), medium inclusions (2.5–5 µm), or small inclusions (<2.5 µm) (n = 3 (mean ± SEM)). Scale bar = 20 µm. *p<0.05, **p = 0.001–0.01, ***p<0.001; Statistics indicate comparison to the disease protein alone for total number of inclusions at each timepoint (black asterisks). Additional statistical comparisons for inclusion size divisions are indicated by color, e.g., dark red asterisk indicates significant change in large inclusions. (**C and D**) With the rh1-GAL4 driver at indicated time points, SDD-AGE and immunoblot analysis show the progression of amyloid formation of MJDtrQ78 and MJDnQ78 proteins over time. Hsp104 did not greatly affect the aggregation profile of MJDtrQ78 but enhanced formation of SDS-insoluble amyloid aggregates of MJDnQ78. Hsp70 suppressed aggregation of both MJD proteins. The formation of large, SDS-insoluble aggregates by SDD-AGE corresponded with the disappearance of SDS-soluble soluble protein from immunoblots. MJDtrQ78 and MJDnQ78 were detected using anti-HA and anti-myc, respectively, with anti-tubulin as a loading control. Band density for both amyloid smears (SDD-AGE) and soluble bands (Western blot) were quantified using ImageJ (n = 3 (mean ± SEM)). *p<0.05, **p = 0.001–0.01, ***p<0.001; Statistics indicate comparison to the disease protein alone at each timepoint.

To probe the mechanism underlying the dichotomous results found for the Hsp104 interaction with MJDtrQ78 and MJDnQ78, an in-depth investigation of the protein aggregates was performed. To slow protein aggregation such that we could analyze underlying protein accumulations in detail, we expressed the transgenes in the eye with an adult-onset driver rhodopsin1(rh1)-GAL4. Analysis of the PolyQ protein accumulations showed that Hsp104 altered the kinetics of inclusion formation for both MJD protein isoforms. By cryosectioning and subsequent immunohistochemistry (IHC), MJDtrQ78 formed compact inclusions that increased in size over time (Fig. 3A, top row). Quantification of inclusion size over time (Fig. 3A, gray bars in graph) reveals distinct inclusion size populations (small <2.5 µm, medium 2.5–5 µm, large >5 µm in optical diameter), demonstrating that inclusions became larger and more numerous with time. Consistent with previous studies, co-expression of Hsp70 delayed the kinetics and significantly reduced MJDtrQ78 protein aggregation (Fig. 3A, bottom row; green bars in graph, p = 0.03). By contrast, Hsp104 initially delayed inclusion formation (p = 0.004, but then significantly enhanced the formation of small inclusions (p = 0.04), eventually reaching accumulation levels similar to that with MJDtrQ78 alone (Fig. 3A, center row; red bars in graph, n.s. p = 0.5).

To examine protein accumulation by biochemical methods, we used SDD-AGE (Semi-Denaturing Detergent–Agarose Gel Electrophoresis), a protein agarose gel technique that can resolve amyloid aggregates [48]. This technique is useful for resolving high molecular weight polymer assemblies that maintain stable contacts in 2% SDS (a feature of highly stable amyloid). SDD-AGE revealed that the truncated MJDtrQ78 protein formed SDS-resistant amyloid structures that accrue with time (Fig. 3C). Unlike Hsp70, which significantly suppressed amyloid formation (p = 0.003), Hsp104 did not change the overall kinetics of MJDtrQ78 amyloid formation or the overall level of aggregation (Fig. 3C). Confirming that insoluble amyloid material was increased, the reduction of SDS-soluble levels of protein by immunoblot matched the concomitant increase in amyloid formation observed by SDD-AGE (Fig. 3C). Thus, Hsp104 rescues MJDtrQ78 toxicity, but the relationship to MJDtrQ78 aggregation is complex. IHC revealed that Hsp104 initially delays MJDtrQ78 inclusion formation, but then significantly enhances the formation of small inclusions (Fig. 3A). However, when amyloidogenesis was tracked by SDD-AGE, Hsp104 affected neither the rate nor the extent of amyloid formation (Fig. 3C). This finding indicates that to rescue toxicity Hsp104 might reduce formation of soluble and toxic oligomeric MJDtrQ78 species that are populated during amyloidogenesis, just as it does with the yeast prion proteins Sup35 and Ure2 [12], [13].

**Figure 4:**
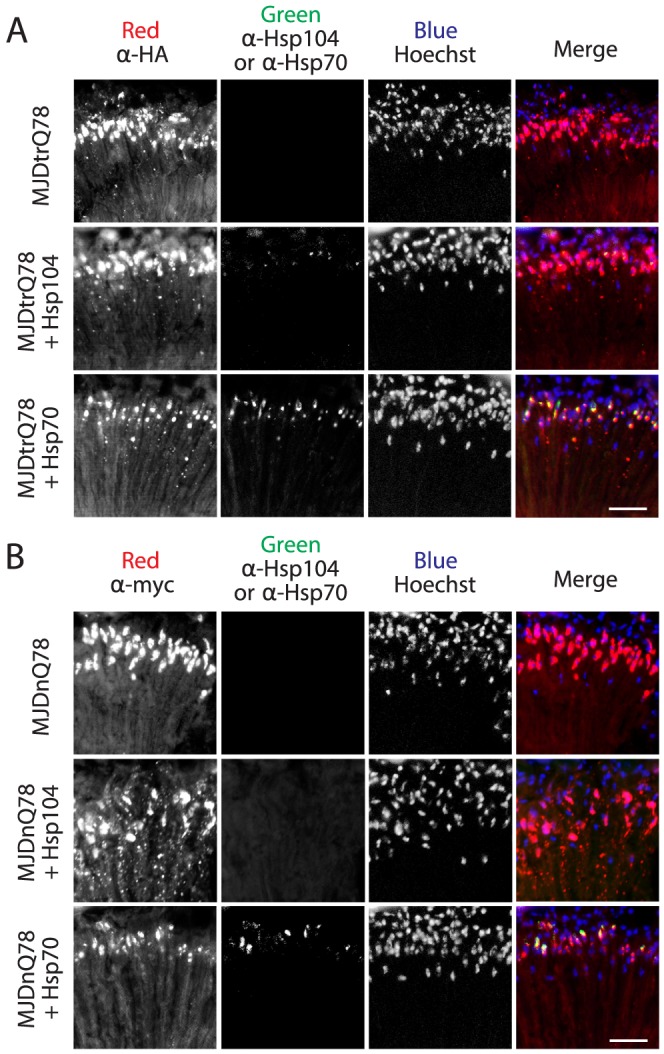
Hsp70, but not Hsp104, colocalizes with both MJDtrQ78 and MJDnQ78 inclusions. (**A**) Split channels from the MJDtrQ78 treatment set at the Day 7 time point (from Fig. 3A). Note that Hsp104 (green, middle row) had minimal overlap with MJDtrQ78 inclusions (red, middle row), but some colocalization was observed. In contrast, Hsp70 (green, bottom row) strongly colocalized with MJDtrQ78 inclusions (red, bottom row). (**B**) Split channels from the MJDnQ78 treatment set at the Day 7 time point (from Fig. 3B). Hsp104 (green, middle row) had no observable colocalization with MJDnQ78 inclusions (red, middle row), while Hsp70 (green, bottom row) did colocalize with MJDnQ78 inclusions (red, bottom row).

Next, we assessed MJDnQ78 misfolding. In contrast to the truncated MJDtrQ78 isoform, the pathogenic full-length MJDnQ78 initially formed amorphous inclusions that did not become more numerous after day 1 (Fig. 3B, top row; gray bars in graph). These MJDnQ78 amorphous aggregates appeared early by IHC, and insoluble amyloid aggregates developed later as observed by SDD-AGE (Fig. 3B and D). Thus, the early MJDnQ78 aggregates are non-amyloid in nature but later convert into the insoluble amyloid structure, closely resembling the two-step aggregation kinetics observed *in vitro* [27]–[29]. As with MJDtrQ78, co-expression of Hsp70 delayed the kinetics of aggregation and significantly suppressed inclusion formation (Fig. 3B, bottom row; green bars in graph, p = 0.02). However, in marked contrast, co-expression of Hsp104 significantly increased the formation of large aggregates at early time points (p = 0.002), and then significantly increased the number of small inclusions over time (Fig. 3B, center row; red bars in graph, p<0.001). Consistent with IHC results, SDD-AGE analysis demonstrated that Hsp104 significantly promoted the early formation of insoluble MJDnQ78 amyloid aggregates (Fig. 3D, p<0.001), whereas Hsp70 delayed kinetics of amyloid formation (Fig. 3D, p = 0.01). Hsp70, but not Hsp104, stably colocalized with both MJDtrQ78 and MJDnQ78 inclusions (Fig. 3A and B; see Fig. 4 for channel breakdown). The striking contrast between the effects of Hsp104 and Hsp70 on inclusion formation reinforces their functional differences [30], [49].

### Domains neighboring the expanded PolyQ tract hinder protective Hsp104 activities

**Figure 5:**
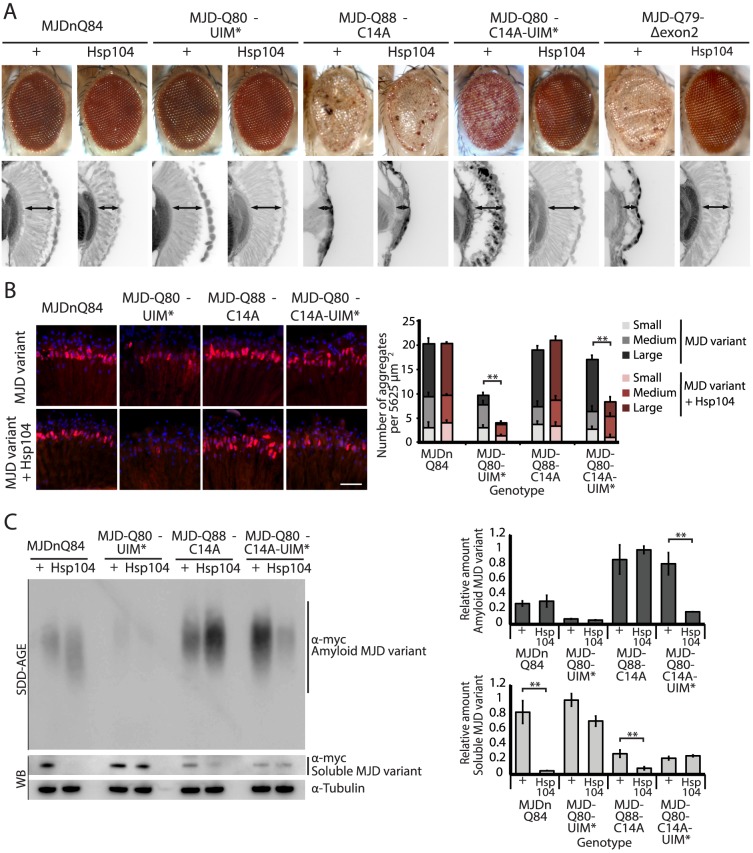
A portion of the Josephin domain and the Ubiquitin-Interacting Motifs prevent Hsp104 from rescuing full-length MJD pathogenicity. (**A**) With the 1×gr-GAL4 driver at d7, external eye and internal retinal structure showed suppression of toxicity by Hsp104 for MJD variants with mutated UIMs (MJD-Q80-UIM* and MJD-Q80-C14A-UIM*). Hsp104 strongly suppressed the external eye degeneration and loss of internal retinal structure of MJD lacking a region spanning the Josephin domain (amino acids 9–63 (Δ exon 2)). The MJDnQ84 and MJD-Q80-UIM* crosses were performed at 29°C to enhance the severity of degeneration. Arrows indicate the width of the retina to highlight changes in tissue integrity. (**B**) With the rh1-GAL4 driver at d3, Hsp104 suppressed inclusion formation in MJD variants with UIM mutations, as seen by IHC (d3). Accumulations of the MJD variant proteins were detected by anti-myc (red) and nuclei are labeled by Hoechst stain (blue). Scale bar = 20 µm. Size of inclusions was quantified using ImageJ, with delineations for large inclusions (>5 µm across), medium inclusions (2.5–5 µm), or small inclusions (<2.5 µm) (n = 3 (mean ± SEM)). Scale bar = 20 µm. **p = 0.001–0.01. (**C**) With the rh1-GAL4 driver at d3, SDD-AGE and Western immunoblot showed that by d3, Hsp104 enhanced aggregation of MJD variants with wild-type UIMs, but reduced formation of amyloid of MJD variants with UIM mutations. MJD variants were detected using anti-myc with anti-tubulin as a loading control. Band density for both amyloid smears (SDD-AGE) and soluble bands (Western blot) were quantified using ImageJ (n = 3 (mean ± SEM)). **p = 0.001–0.01.

**Figure 6:**
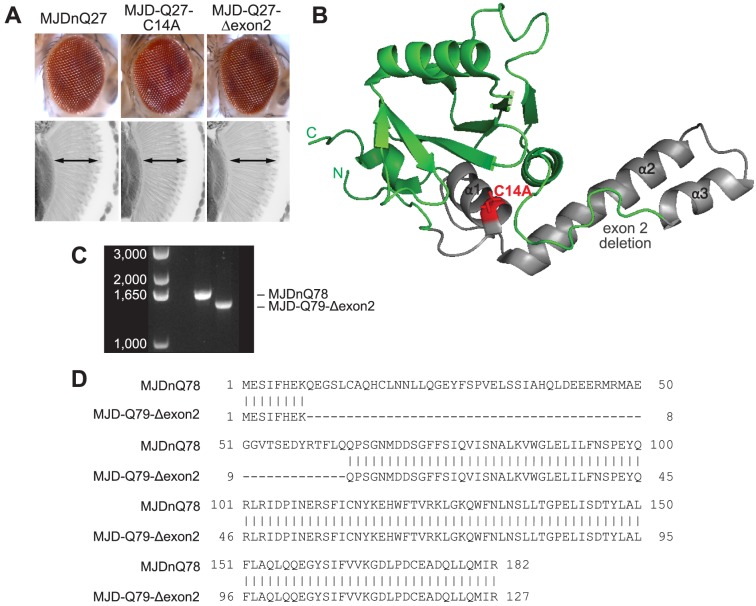
Characterization of DUB-deficient MJD variants. (**A**) Driven by 1×gr-GAL4 at d7, MJD with non-expanded Q has no toxicity. Loss of DUB activity through point mutation, MJD-Q27-C14A, or through exon deletion, MJD-Q27-Δexon2, did not confer toxicity. (**B**) The structure of the Josephin domain, from PDB file 1YZB. The amino acids lost in the exon 2 deletion are highlighted in gray and the catalytic residue mutated in the C14A variant is highlighted in red. (**C**) Genomic DNA from *Drosophila* was amplified for the UAS insert and was resolved on an agarose gel. We confirmed that MJD-Q79-Δexon2 was missing the appropriate size of DNA contained within exon 2. (**D**) Sequencing of the Josephin domain confirmed that MJD-Q79-Δexon2 lacked the bases encoding amino acids 9–63, but was otherwise identical to MJDnQ78.

**Figure 7:**
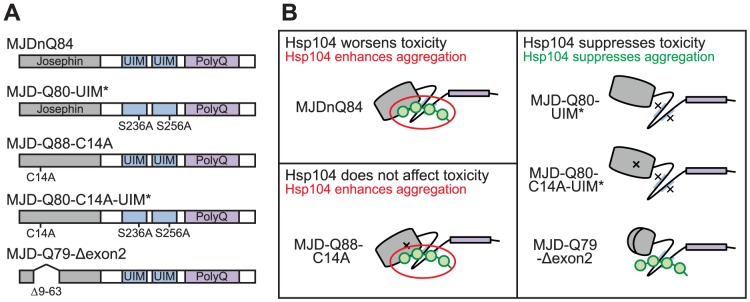
Model of MJD domain contribution to the interaction with Hsp104. (**A**) Schematic of MJD variants with functional deficiencies. (**B**) Model of MJD protein conformation as it affects accessibility to Hsp104 treatment. Hsp104 worsens the pathogenicity and enhances aggregation of MJD variants with functional UIMs and an intact Josephin domain. A closed loop may be formed between these domains, potentially through mutual association with a poly-ub chain, which inhibits productive remodeling by Hsp104. MJD variants with a more flexible conformation are receptive to successful remodeling by Hsp104.

While it is known that in select conditions, Hsp104 promotes amyloid formation of specific yeast prions [12], [13], [30], we did not anticipate that Hsp104 would have opposite actions on two constructs of the same PolyQ protein. Thus, we assessed which domains of the full-length MJD protein prevented rescue by Hsp104 by employing a series of expression-matched MJD variants with disruptions to specific motifs (Fig. 5, 6, and see summary in Fig. 7A). Because the PolyQ domains are pure CAG repeats, they are subject to instability. Given this instability, the repeat lengths have been matched as closely as possible with matching protein expression levels (see [41]). Because of the reduced expression level by the 1×gr-GAL4 driver used for these experiments, MJDnQ84 (the pathogenic protein for this set of expression matched proteins) now showed mild degeneration. However, as before, analysis of retinal integrity demonstrated that the toxicity of MJDnQ84 was enhanced upon co-expression of Hsp104 (Fig. 5A). In contrast, an MJD variant in which both UIMs were mutated and unable to engage poly-ub, MJD-Q80-UIM* (Fig. 7A), exhibited mild toxicity that was suppressed by Hsp104 (Fig. 5A). Thus, the ability of the UIM domains to engage poly-ub hinders protective Hsp104 activity.

We also examined variants lacking DUB activity through mutation of the active site in the Josephin domain, MJD-Q88-C14A (Fig. 7A), which causes more severe toxicity than MJDnQ84 due to the loss of the physiological UPS function [41]. This occurs because DUB activity of MJD can suppress its own PolyQ toxicity; the C14A mutation is innocuous when MJD has a normal length, non-expanded Q repeat (Fig. 6A) [41]. Co-expression of Hsp104 did not affect the severe MJD-Q88-C14A toxicity (Fig. 5A). However, when the active site mutation was combined with the UIM mutations, in MJD-Q80-C14A-UIM* (Fig. 7A), Hsp104 now suppressed toxicity (Fig. 5A). This result reiterates that functional UIMs hinder rescue by Hsp104. We further examined a separate splice variant lacking DUB activity through an exon deletion that includes the active site, MJD-Q79-Δexon2 (missing amino acids 9–63) (Fig. 6B, 7A) [50], [51], which, like MJD-Q88-C14A, conferred severe toxicity (Fig. 5A). Co-expression of Hsp104 with MJD-Q79-Δexon2 strongly suppressed degeneration (Fig. 5A). Thus, Hsp104 mitigated MJD toxicity when the exon containing the active site was deleted (MJD-Q79-Δexon2) but had no effect when the active site was inactivated by a single point mutation (MJD-Q88-C14A). Taken together, these data indicate that functional UIMs and an intact Josephin domain both prohibit Hsp104 from rescuing full-length MJDnQ84 toxicity.

To uncover additional mechanistic insight into the interactions with Hsp104, we examined inclusion formation and kinetics with adult-onset rh1-GAL4 expression. By IHC, the MJD variants formed accumulations in a manner roughly consistent with severity of eye degeneration (Fig. 5B, top row; gray bars in graph). Those variants with mutated UIMs, MJD-Q80-UIM* and MJD-Q80-C14A-UIM*, showed significantly reduced levels of aggregate formation with Hsp104 (Fig. 5B, bottom row; red bars in graph, p = 0.003 for both). SDD-AGE analysis revealed that Hsp104 significantly enhanced the conversion of soluble protein to SDS-resistant polymers for variants with intact UIMs: MJDnQ84 and MJD-Q88-C14A (Fig. 5C, p = 0.002 and p = 0.005, respectively). Moreover, Hsp104 significantly reduced the formation of amyloid material by MJD-Q80-C14A-UIM* (Fig. 5C, p = 0.01). The MJD-Q79-Δexon2 protein was not detectable by immunoblot (but was confirmed by genotyping, Fig. 6C, D), precluding aggregate analysis of this variant. These findings underscore the role of active ubiquitin binding in obstructing productive remodeling by Hsp104.

A summary of the effect of Hsp104 on MJD variants is presented in Fig. 7B. In the two cases in which Hsp104 enhanced aggregation (MJDnQ84, MJD-Q88-C14A), the MJD protein has both functional UIMs and an intact Josephin domain. By contrast, protein variants whose toxicity and underlying protein accumulations were suppressed by Hsp104 (MJD-Q80-UIM*, MJD-Q80-C14A-UIM*, MJD-Q70-Δexon2) each lack UIM binding or a portion of the Josephin domain. This supports a model in which an inflexible or “closed” loop is formed, possibly through associations with a poly-ub chain, between the functional UIMs and the intact Josephin domain (Fig. 7B, red circle). Our hypothesis is that Hsp104 is able to effectively remodel a more flexible or “open” conformation of select protein variants (e.g., MJD-Q80-UIM*, MJD-Q80-C14A-UIM*, MJD-Q70-Δexon2, or the truncated MJDtrQ78), but that the inflexible/closed conformation of other proteins (e.g., MJDnQ78, MJDnQ84, or MJD-Q88-C14A) obstructs protective Hsp104 activities.

### Active remodeling by Hsp104 is required for modulation of protein pathogenicity

**Figure 8:**
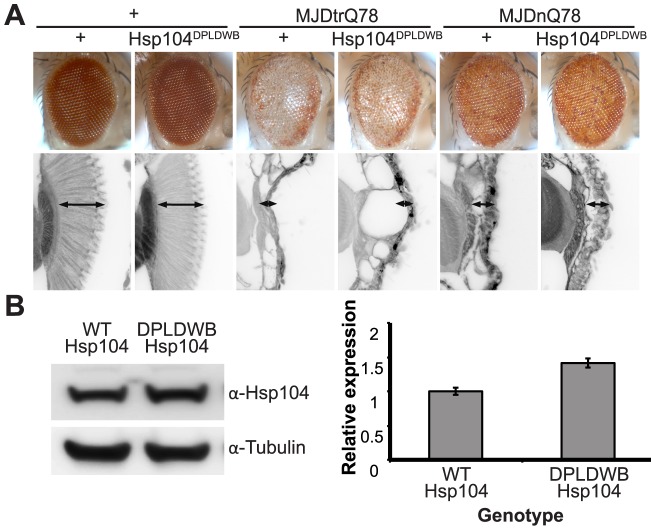
ATPase activity and substrate binding are required for Hsp104 to modulate disease. (**A**) With the gmr-GAL4 driver at d7, expression of the inactive mutant UAS-Hsp104^DPLDWB^, which is unable to bind substrate or hydrolyze ATP, caused no effect on its own when expressed by gmr-GAL4. Additionally, the inactive Hsp104^DPLDWB^ did not modulate the toxicity of MJDtrQ78 or MJDnQ78. Eye images and retinal sections showed moderate degeneration upon expression of MJDtrQ78 or MJDnQ78, but unlike wild-type Hsp104 (see Fig. 2), Hsp104^DPDLWB^ did not mitigate the degeneration caused by MJDtrQ78 nor did it enhance the toxicity of MJDnQ78. Arrows indicate the width of the retina to highlight changes in tissue integrity. (**B**) Western immunoblot demonstrated that WT Hsp104 and Hsp104^DPLDWB^ had similar expression levels. Tubulin served as a loading control. Quantification of Western immunoblots confirmed that protein expression levels were similar, but that Hsp104^DPLDWB^ was expressed at levels slightly higher than WT Hsp104. Hsp104 signal was normalized to tubulin (n = 3 (mean ± SEM)).

To verify the critical role of active remodeling by Hsp104, we created an ATPase-Dead and substrate-binding defective Hsp104 transgenic fly. We introduced four mutations (Y257A:E285Q:Y662A:E687Q) into Hsp104 to ensure that Hsp104 could not engage substrate or hydrolyze ATP, creating the mutant known as Double Pore Loop Double Walker B (Hsp104^DPLDWB^), which is structurally identical to wild-type but functionally inactive [5]. Unlike wild-type Hsp104, which caused mild retinal disruption by the gmr-GAL4 driver, similar expression levels of Hsp104^DPLDWB^ were innocuous (Fig. 8A, B). Moreover, Hsp104^DPLDWB^ did not modulate the toxicity of either MJDtrQ78 or MJDnQ78 (Fig. 8A), underscoring the importance of substrate translocation for Hsp104 to mitigate MJDtrQ78 or worsen MJDnQ78-associated degeneration. Thus, ATPase activity and substrate binding are required *in vivo* for modulatory effects of Hsp104.

### Hsp104 suppresses progression of pre-existing degenerative disease *in vivo*

**Figure 9:**
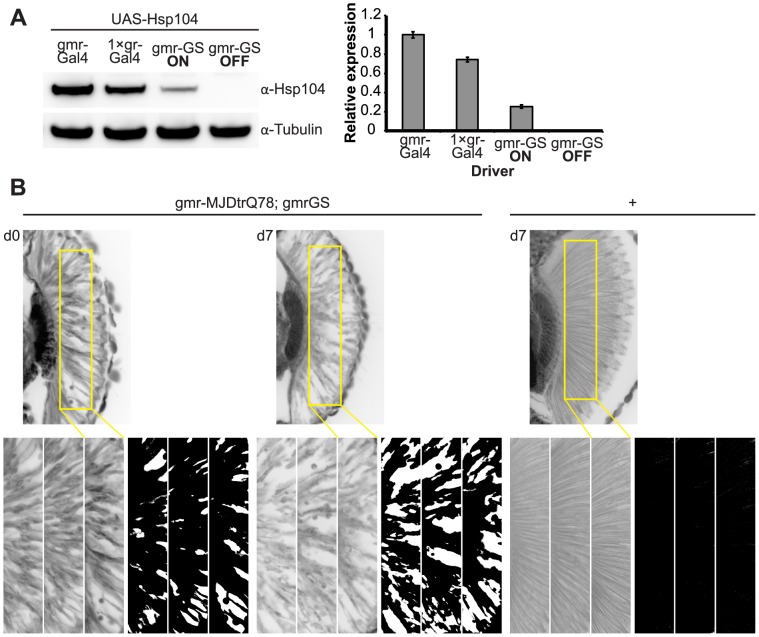
Establishing the GeneSwitch paradigm. (**A**) Western immunoblot demonstrated that the gmr-GS-GAL4 line specifically drove Hsp104 expression in the presence of RU486 (gmr-GS ON), but there was no expression of Hsp104 in the absence of the drug (gmr-GS OFF) at d7. The level of expression by gmr-GS was lower than with the other eye-specific drivers gmr-GAL4 and 1×gr-GAL4. Tubulin served as a loading control. Quantification of Western immunoblots confirmed that gmr-GS expressed at a lower level than the other drivers, with the amount of Hsp104 expressed by gmr-GS reaching about 33% of that expressed by 1×gr-GAL4. Hsp104 levels were normalized to tubulin (n = 3 (mean ± SEM)). (**B**) Paraffin sections demonstrate that the retinal tissue loss associated with gmr-MJDtrQ78 is apparent at d0, and progresses through d7. In comparison, control flies (7 d) display no such loss of retinal integrity. For each example shown here, a 7,000 µm^2^ rectangular selection (used for quantification in Fig. 10) of a retinal section from three independent animals is presented. Each region was converted to a black and white image to show the area covered by tissue and quantitated by ImageJ analysis (see Methods). For the analysis in Fig. 10, regions from 6 independent animals were used for quantitation; all experiments were repeated at least three times with similar results.

Hsp104 is unique in its capacity to reverse pre-existing amyloids in yeast and *in vitro* [5], [52]. However, the potential of Hsp104 to affect pre-existing protein-aggregation disease in a metazoan, *i.e.*, a genuine *in vivo* treatment situation, has never been addressed. To address this deficit, we constructed fly lines containing three elements: (1) the toxic MJDtrQ78 protein driven directly by a gmr element such that the disease-associated protein was constitutively expressed in the eye; (2) a drug-inducible gmr-GAL4 driver known as “GeneSwitch” (gmr-GS) to activate GAL4 expression only in the presence of the drug RU486 (mifepristone) [53], [54]; and (3) the UAS-HSP treatment molecule (here, Hsp104 or Hsp70), such that the HSP will be expressed conditionally only when RU486 is present in the fly food (Fig. 9A). This system allows the activation of HSP expression sequential to disease-associated protein onset. In this manner, we could test the ability of exogenous HSPs to mitigate the toxicity of the pathogenic PolyQ protein after the pathogenic protein was already accumulating in aggregated forms and causing degeneration. We hypothesized that, due to its disaggregation rather than chaperone activity, Hsp104 may have the potential to markedly mitigate degeneration associated with pre-existing PolyQ protein aggregates.

**Figure 10:**
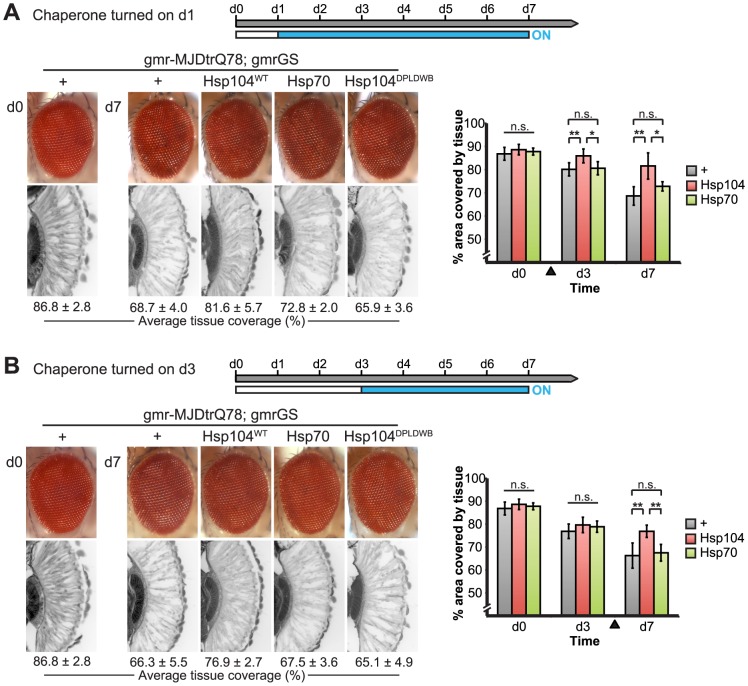
Progressive MJDtrQ78 pathogenicity can be suppressed by expression of Hsp104 after onset of degeneration. (**A**) External eye and internal retinal sections demonstrate the effect of sequential onset of chaperone treatment. The adult animals emerged from the pupal case with a disrupted eye (d0); chaperone activity was initiated at d1. Disruption caused by MJDtrQ78 was significantly suppressed by activation of Hsp104 at d1. In contrast, Hsp70 or the inactive Hsp104^DPLDWB^ did not significantly impact the progression of pathology. To quantify degeneration within the retina, the percentage of a standard area covered by tissue was measured by ImageJ (n = 6 (mean ± SD)) (see also Supplemental Figure 7). *p<0.05, **p = 0.001–0.01. (**B**) The progression of toxicity of MJDtrQ78 was also significantly altered by sequential activation of Hsp104 later in the degenerative process, on d3. Quantification of tissue as above (n = 6 (mean ± SD)). **p = 0.001–0.01.

We established that retinal degeneration associated with gmr-MJDtrQ78 had begun at the time of adult fly emergence (d0) and progressed in severity to d7 (Fig. 9B, Fig. 10). We then activated Hsp104 or Hsp70 expression at an early time point (d1), or a later time point (d3), and examined the pathogenic impact of the MJDtrQ78 protein at d7 by retinal section. When activated at d1, Hsp104 was able to significantly mitigate retinal degeneration associated with MJDtrQ78 (p = 0.001), while Hsp70 did not have a significant effect (Fig. 10A, n.s. p = 0.06). These data show that Hsp104 is significantly more effective than Hsp70 at mitigating toxicity once disease progression has begun (Fig. 10A, p = 0.01). Importantly, inactive Hsp104^DPLDWB^ had no effect (Fig. 10A). Hsp104 significantly improved tissue structure even when expression was induced at a later time point of d3 when degeneration was even more severe (Fig. 10B, p = 0.003). Moreover, while MJDtrQ78 treated with Hsp70 continued to degenerate with time, induction of Hsp104 arrested disease progression (Fig. 10A, d7 vs d1; Fig. 10B, d7 vs d3). Thus, Hsp104 mitigates pathogenesis even when administered *after* the onset of pathogenic protein-induced degeneration.

**Figure 11:**
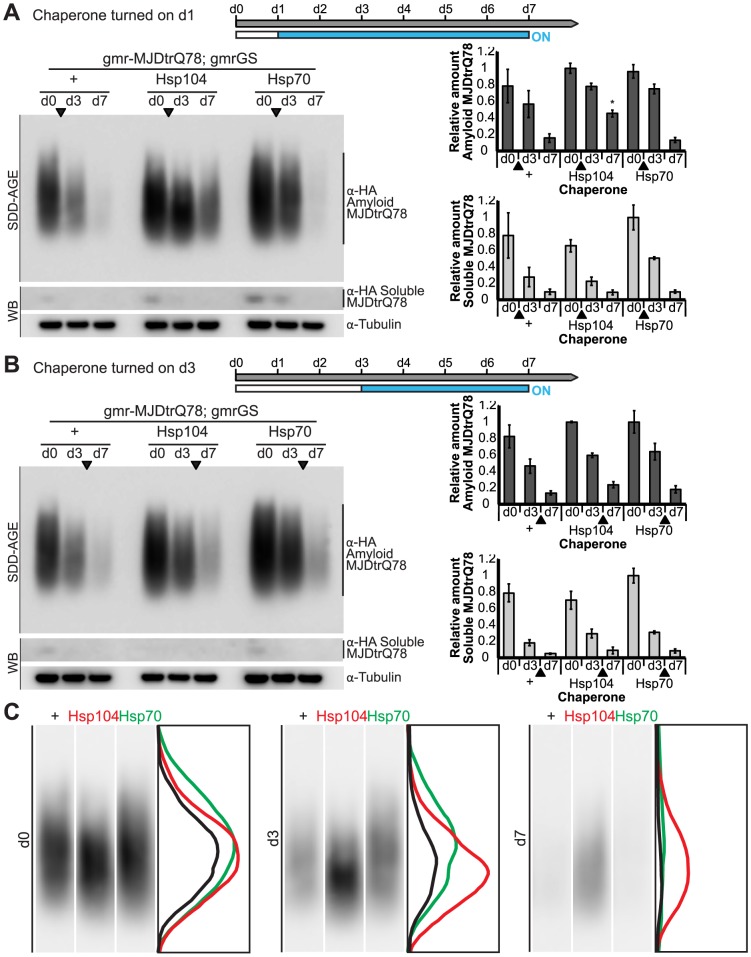
Hsp104 does not mitigate disease progression by clearing MJDtrQ78 amyloid fibrils. (**A** and **B**) SDD-AGE and immunoblot analysis show the progression of amyloid formation of gmr-MJDtrQ78 over time. Drug activation of disaggregase or chaperone at the early time point of d1 (**A**) shows that induced Hsp104 expression significantly increased amyloid load by d7. Hsp70 had no significant impact on amyloid levels. Activation of the molecules at later time point d3 (**B**) demonstrated that neither Hsp104 nor Hsp70 affected amyloid load. MJDtrQ78 was detected using anti-HA antibody with anti-tubulin as a loading control. Band density for both amyloid smears (SDD-AGE) and soluble bands (Western blot) were quantified using ImageJ (n = 3 (mean ± SEM)). *p<0.05; Statistics indicate comparison to the disease protein alone at each timepoint. (**C**) Representative densitometry traces of the SDD-AGE amyloid immunoblots, as in A, show the distribution of amyloid species resolved by size. After HSP activation at d1, the distribution of MJDtrQ78 amyloid accumulations exposed to Hsp104 shifts downward by d3 (red trace), compared to the control (black) and Hsp70 (green) profiles. The densitometry analysis was performed in ImageJ and the effect was reproducible among three independent replicates.

Next, we examined the underlying protein aggregates by SDD-AGE and Western immunoblot. We observed that gmr-MJDtrQ78 had high levels of amyloid, and this was lessened with time (potentially due to tissue loss) (Fig. 11). When turned on at d1, Hsp104 did not reverse MJDtrQ78 amyloid formation, but rather significantly increased the amyloid present by d7 (p = 0.02), while Hsp70 had no effect (Fig. 11A). Neither molecule significantly altered amyloid load when turned on at d3 (Fig. 11B). These results imply that Hsp104 is not acting as a MJDtrQ78-amyloid disaggregase, but rather is mitigating toxicity in a distinct manner, which is also consistent with our earlier results where MJDtrQ78 and Hsp104 are expressed at the same time (see Fig. 2, 3). Intriguingly, representative densitometry traces for the amyloid smears for each treatment condition turned on at d1 suggest that the peak of MJDtrQ78 amyloid species shifts downward to indicate smaller amyloid accumulations by d3 following Hsp104 activation (Fig. 11C). No shift in the densitometry trace is observed for the control or Hsp70 treatment (Fig. 11C), suggesting that Hsp104 is indeed altering the character of amyloid species although not eliminating these fibrils completely. In summary, this novel system of temporally controlled HSP expression demonstrates that although concomitant expression of Hsp70 is more successful than Hsp104 at preventing degeneration (see Fig. 2), inducible expression of Hsp104 is more effective than Hsp70 at suppressing disease progression once protein aggregation and degeneration are already established (Fig. 10).

## Discussion

Here, we reveal key novel insights into the efficacy and interactions of Hsp104 with the pathogenic PolyQ protein MJD. Our studies reveal the surprising finding that Hsp104 interacts differentially with different forms of the MJD protein. Hsp104 is a potent suppressor of toxicity of the truncated protein, but an enhancer of toxicity of the full-length protein. These differences are determined by specific domains of MJD that are not directly implicated in aggregation. Our findings have also uncovered a heretofore unrecognized and important application of Hsp104 *in vivo*, which is its ability to mitigate the course of protein-aggregation disease even after it has already initiated. Indeed, our studies show that Hsp104 is able to mitigate disease progression once it has begun, unlike the classical metazoan chaperone Hsp70. These studies provide new insight into the *in vivo* effects of Hsp104 in the context of a therapeutic agent.

### Hsp104 has opposite effects on two constructs of the same disease protein

Our detailed investigations of the effects of Hsp104 on the MJD protein led to the unexpected result that Hsp104 has opposite effects on the toxicity of different versions of the MJD protein (MJDtrQ78 and MJDnQ78), despite the fact that these proteins contain the same pathogenic PolyQ stretch. These disparate actions indicate that Hsp104 might be a useful probe to understand the nature of aggregates and the toxicity imposed by them. Hsp70 suppressed both MJDtrQ78 aggregation and toxicity (Fig. 2E, 3C). By contrast, Hsp104 mitigated toxicity of MJDtrQ78 without suppressing the extent of MJDtrQ78 aggregation (Fig. 2C, 3C). This result suggests that MJDtrQ78 aggregation per se need not be deleterious. Uncoupling of aggregation and toxicity has also been observed in other settings. For example, numerous genetic suppressors of FUS and TDP-43 toxicity, which are connected with amyotrophic lateral sclerosis and frontotemporal dementia, rescue toxicity without affecting FUS or TDP-43 aggregation in yeast [55]–[57]. We suggest that Hsp104 likely mitigates toxicity of MJDtrQ78 accumulations via subtle biochemical changes rather than gross changes in aggregation levels.

What might these biochemical changes be? Hsp104 can disrupt toxic soluble oligomers of various proteins, including Sup35, which may help explain why Sup35 prion formation is not intrinsically toxic to yeast [5], [12], [13], [31], [58]. Thus, Hsp104 might eliminate toxic soluble oligomers formed by MJDtrQ78 just as it does for Sup35 and alpha-synuclein [5], [12], [13], [31]. Furthermore, the amyloid-remodeling activity of Hsp104 can selectively amplify some amyloid strains (i.e. different cross-β structures formed by the same polypeptide) at the expense of others [59]. Given that PolyQ can access both toxic and benign amyloid strains [60], it is plausible that the presence of Hsp104 might amplify benign amyloid strains of MJDtrQ78 at the expense of toxic strains [59], [60]. Indeed, we observed that Hsp104 activity visibly altered the distribution of MJDtrQ78 amyloid species (Fig. 11C) suggesting that Hsp104 may be selectively eliminating certain fibril strains. Finally, to promote toxicity amyloid structures typically sequester large metastable proteins with unstructured regions, which occupy key nodes in functional networks linked to transcription, translation, chromatin organization, cell structure, and proteostasis [61], [62]. Hsp104 might disaggregate and rescue these proteins sequestered by MJDtrQ78 amyloid or promote the formation of MJDtrQ78 amyloid strains that do not deplete such an essential constellation of proteins. Further studies are needed to distinguish these non-mutually exclusive possibilities.

In other settings, it has been suggested that chaperone-initiated formation of large, insoluble amyloid aggregates can actually be protective by sequestering potentially toxic pre-fibrillar conformers [63]–[65]. Our results, however, indicate that, at least for Hsp104-driven enhancement of MJDnQ78, increased and accelerated aggregation is more toxic than MJDnQ78 aggregation that occurs in the absence of Hsp104. Our findings illustrate the complex relationship between aggregation and toxicity, which likely extends to other neurodegenerative disease models [64]. Moreover, our studies suggest that cautious interpretation is required when translating findings from cell culture experiments to neurodegeneration in animal models [34], [66]. Although Hsp104 is not found in the metazoan proteostasis network, our observations could help inform how to manipulate existing components of the metazoan proteostasis network for therapeutic purposes. Thus, components that suppress MJDnQ78 aggregation are likely beneficial, whereas MJDtrQ78 toxicity can be mitigated without having to suppress MJDtrQ78 aggregation.

Moreover, our findings demonstrate that an agent with a mitigating effect on the truncated version of the MJD protein may act in a different manner against the full-length MJD protein. Thus, what is good for one may not be beneficial for the other. In MJD/SCA3, as well as other neurodegenerative diseases, fragmentation of the disease protein may initiate aggregation and this process is critical for disease progression [42], [43], [67], [68]. Therefore, agents that effectively eliminate one specific sub-population of toxic protein accumulation but enhances another toxic sub-population may not be therapeutically viable in the complicated mixed populations that occur in disease. Our results highlight the complexity in developing therapeutic agents for neurodegenerative disorders.

### Protein context is critical in evaluating disease-associated proteins

Although Hsp104 enhances MJDnQ78 amyloidogenesis and toxicity, we found that elimination of functional domains not implicated in PolyQ aggregation facilitated the ability of Hsp104 to suppress MJD-associated degeneration. Elimination of UIM functionality or removal of a component of the Josephin domain (exon 2) restored the remodeling capacity of Hsp104. This suggests that MJDnQ78 pathogenicity is not intrinsically intractable, but is capable of being suppressed by Hsp104 if other domains of the protein are inactivated (e.g., the UIMs). Alternatively, potentiated or MJDnQ78-optimized Hsp104 variants might be developed that are able to overcome these hindrances via increased unfolding power [5], [17].

Our studies underscore the importance of protein context in studying protein-misfolding diseases. Within the protein itself, neighboring domains not thought to be involved in aggregation may be impacting accumulation kinetics and the biochemical properties of inclusions [69], as well as accessibility of the aggregation domain to potential disaggregase therapeutics. That Hsp104 efficiently mitigates toxicity of MJD variants with ubiquitin-binding defects also demonstrates that, in addition to protein context, the cellular context of the protein is critical to consider; for example, the interaction between poly-ub chains and MJD may hinder protective Hsp104 modalities.

Previous studies *in vitro* have characterized aggregation of the full-length, pathogenic MJD protein as a two-step process in which the protein assembles first into SDS-soluble fibrillar polymers associating via the Josephin domain, and then converts to SDS-insoluble amyloid fibers driven by the PolyQ domain [27]–[29], [70]. We propose that this two-step process occurs *in vivo* as well. Indeed, it is consistent with our observation that full-length MJDnQ78 forms amorphous accumulations that appear visually by IHC before they can be observed as SDS-insoluble amyloid aggregates by SDD-AGE (see Fig. 3B and D). We suspect that full-length MJD initially forms SDS-soluble, Josephin-driven non-amyloid accumulations that initiate Hsp104 remodeling.

The initial formation of non-amyloid polymers is also compatible with our model of Hsp104 interacting differentially with the “open” and “closed” conformations of the protein discussed above (see Fig. 7B). We hypothesize that a poly-ub chain creates the closed loop by mutually interacting with the UIMs and the flexible helical hairpin encoded by exon 2 in the Josephin domain (Fig. 6B), as this arm is thought to be important for interacting with substrates [71]. Further experiments are required to confirm a poly-ub-mediated interaction between the two domains. According to our model, Hsp104 is able to efficiently translocate and release proteins that are more flexible (e.g., MJD-Q80-UIM*), resulting in fewer aggregates (see Fig. 5B). But due to an inflexible conformation imposed by the UIMs and the Josephin domain, Hsp104 is unable to efficiently remodel proteins containing the closed loop (e.g., MJDnQ84). This incomplete or slow translocation may expose or “prime” the PolyQ region to drive the formation SDS-insoluble amyloid inclusions (see Fig. 3D, Fig. 5C).

Our model suggests that the UIMs and the Josephin domain act together to obstruct Hsp104 remodeling, but we cannot rule out a separate function of the Josephin domain outside of poly-ub interactions. For example, removal of 55 amino acids might destabilize the Josephin domain such that it gets proteolytically cleaved and Hsp104 would then encounter a protein similar to MJDtrQ78 and rescue toxicity. Alternatively, because the MJD-Q79-Δexon2 protein could not be detected by biochemical methods, we cannot exclude the possibility that the deletion within the Josephin domain disrupts the proposed process of Josephin-domain-driven polymerization. In this case, toxicity of this variant may be dependent on highly soluble, possibly oligomeric species, which are effectively targeted by Hsp104.

These findings indicate that there is an opportunity to tailor therapies that are optimized for a specific disease scenario. In the case of full-length MJD, if inefficient translocation by Hsp104 does indeed drive the switch from less toxic SDS-soluble aggregates to highly toxic SDS-insoluble amyloid inclusions, then the development of substrate-optimized Hsp104 mutants (or Hsp104 mutants with altered ATPase rates or unfolding power) may increase efficiency of such interactions and enable Hsp104 to rescue disease phenotypes. Moreover, if UIM binding to poly-ub chains is impairing access of Hsp104 to MJD, this suggests that co-administering an agent to modulate function of a neighboring domain may affect the access of a treatment to the aggregation-prone domain. Indeed, increasing global DUB activity coupled with Hsp104 induction could overcome antagonism due to poly-ub chains.

### Implications for Hsp104 as a therapeutic agent

Chaperone treatment, and examination of Hsp70 in particular, has been an exciting avenue of research in the battle to combat and contain neurodegenerative disease [72], [73]. However, all studies investigating Hsp70 as a modulator of disease have looked only at the chaperone transgenically co-expressed or activated prior to the disease insult. In a study seeking to activate existing Hsp70 rather than introducing exogenic expression, researchers sought to evaluate the chaperone in a mouse model of PD by boosting endogenous Hsp70 expression by treating animals with geldanamycin [74]. While this procedure has the potential to test true reversal of disease course, the authors found that beneficial effects were only observed if upregulation of Hsp70 was initiated prior to pharmacological induction of the PD phenotype [74]. In fact, in a cell culture PD model, geldanamycin was required at least 24 hours prior to disease protein transfection to provide any protection against inclusion formation [75]. In addition, pharmacologic activation of Hsp70 has been shown to suppress both PD and PolyQ disease in *Drosophila* [76], [77], but again, these manipulations were performed prior to disease onset. Despite the existing pharmacological paradigms, and other genetic tools available, such as the tetracycline-inducible system in mouse, no group has evaluated specific chaperone or disaggregase expression induced subsequent to expression of a disease-associated protein.

An inducible system is particularly well suited for Hsp104 because of its unique ability to rapidly dismantle pre-existing amyloid aggregates. Since metazoan chaperones can only very slowly depolymerize amyloid [16], Hsp104 may be more effective in an environment with pre-existing aggregation than a chaperone such as Hsp70, which is more adapted to prevent the initial aggregation. Here, we address the value of temporally controlled induction of disaggregase function after the initiation of PolyQ protein aggregation and the beginning of disease progression. To our knowledge, previous research has been performed with concomitant expression of a therapeutic gene, and thus does not distinguish prevention of disease from halting the progression of the disease state. Neurodegenerative diseases are not detected until later in life, and symptoms may not be apparent until pathological damage has accumulated beyond a tolerable point [78]–[80]. Thus, an agent that can rapidly impact the existing trajectory would be more valuable than one that can only prevent the development of the disease.

Our experimental paradigm offers the exciting possibility to address the efficacy of Hsp104 (or other molecules) in a more genuine therapeutic setting. Indeed, we found that turning on Hsp104 was able to significantly suppress disease-associated degeneration. Interestingly, however, Hsp104 did not disaggregate MJDtrQ78 amyloid in these experiments (Fig. 11A, B). Thus, Hsp104 might mitigate disease progression in this setting by: (a) eradicating toxic soluble MJDtrQ78 oligomers, (b) amplifying benign amyloid forms of MJDtrQ78 at the expense of toxic MJDtrQ78 amyloid, (c) by disaggregating and rescuing essential metastable proteins sequestered by MJDtrQ78 aggregates. Our observation that activation of Hsp104 shifted the MJDtrQ78 amyloid smears resolved by SDD-AGE toward smaller species without eliminating the total amyloid population (Fig. 11C) suggests that Hsp104 may indeed have strain selectivity. Further studies are required to define precisely how Hsp104 mitigates disease progression. Our data show that Hsp70 induction after MJD-associated degeneration has already initiated was unable to significantly mitigate disease progression. Optimization of Hsp70 expression, or administration of the suite of chaperones (for example, Hsp70 with Hsp110 and Hsp40), may improve the outcome, but our findings are consistent with other reports that Hsp70 must be administered before disease initiation to have a positive effect [74], [75]. Our observation that even a later-onset induced expression of Hsp104 is able to significantly suppress progressive PolyQ degeneration suggests that it is possible to mitigate disease phenotypes even after aggregates have begun accumulating and marked pathological degeneration is underway.

Naturally, several barriers must be surmounted to translate Hsp104 into a therapeutic agent for human neurodegenerative disease [17], [18]. Not least is the issue that gene therapy might be required to introduce Hsp104 (or any other genetic modifier) as a therapeutic agent. Gene therapy has yielded encouraging preclinical results for several disorders including congenital blindness [81]–[83]. However, technical and safety issues restrict facile translation to the clinic. Indeed, gene therapy for neurodegenerative diseases remains in early developmental stages and considerable caution is essential at this time. However, initial studies have generated cautious optimism that gene therapy in the adult brain might be safe for various neurodegenerative disorders, including Parkinson's disease [84]–[88]. Thus, even though we await several key advances before any Hsp104 gene therapy (or any other gene therapy) becomes truly viable it is, nonetheless, important to develop solutions to protein misfolding and to test these solutions both *in vitro* and in the most appropriate animal models. Moreover, the fact that Hsp104 is well tolerated by mammalian systems is encouraging [31], [32], [34]–[36], [66]. Ultimately, we envision that only transient expression of Hsp104 (or a substrate-optimized variant) would be required to provide therapeutic benefit. In this way, long-term expression of an exogenous agent and potential off-target side effects would be minimized. Alternatively, methods could be developed to deliver pure Hsp104 (or a substrate-optimized variant) to targeted areas in a single or multiple doses, and thereby avoid issues connected with long-term expression. These various issues and others highlight the complexities of designing therapeutics to treat human neurodegenerative disease.

Finally, the concept of using a yeast protein as the basis for a therapeutic agent might at first glance seem implausible. However, it must also have seemed equally implausible to use a lethal protein toxin from the bacterium, *Clostridium botulinum*, as a therapeutic agent. Despite being a deadly toxin, botulinum toxin variants have found key clinical applications due to their highly potent and selective ability to cleave SNARE proteins and prevent secretion [89]. Importantly, they are used to treat a variety of neuromuscular disorders including: blepharospasm, strabismus, muscle spasms, upper motor neuron syndrome, cervical dystonia and chronic migraine [90]–[95]. Indeed, the massive clinical success of botulinum toxin variants suggests it is critical to identify potentially therapeutic biological activities that originate in the microbial world and utilize and develop them to treat human disease.

## Materials and Methods

### *Drosophila* transgenic lines and crosses

Transgenic flies expressing UAS-Hsp104 and UAS-Hsp104^DPLDWB^ were generated by standard techniques using the pUAST vector [38]. In order to boost expression of the transgene, pUAST-Hsp104 was codon-optimized (via gene synthesis; GenScript) for expression in *Drosophila* and a Kozak sequence (ACAAA) was added prior to the start codon [37].

The full sequence of codon-optimized Hsp104 is:

ATGAACGATCAGACCCAGTTCACCGAGCGCGCCCTGACCATCCTGACCCTGGCCCAGAAGCTGGCCAGCGATCACCAGCACCCCCAGCTGCAGCCCATCCACATCCTGGCCGCCTTCATCGAGACCCCCGAGGATGGCAGCGTGCCCTACCTGCAGAACCTGATCGAGAAGGGCCGCTACGATTACGATCTGTTCAAGAAGGTGGTGAACCGCAACCTGGTGCGCATCCCCCAGCAGCAGCCAGCCCCAGCCGAGATCACCCCAAGCTACGCCCTGGGCAAGGTGCTGCAGGATGCCGCCAAGATCCAGAAGCAGCAGAAGGATAGCTTCATCGCCCAGGATCACATCCTGTTCGCCCTGTTCAACGATAGCAGCATCCAGCAAATCTTCAAGGAGGCCCAGGTGGATATCGAGGCCATCAAGCAGCAGGCCCTGGAGCTGCGCGGAAACACCCGCATCGATAGCCGCGGAGCCGATACCAACACCCCCCTGGAGTACCTGAGCAAGTACGCCATCGATATGACCGAGCAGGCCCGCCAGGGAAAGCTGGACCCAGTGATCGGACGCGAGGAGGAGATCCGCAGCACCATCCGCGTGCTGGCCCGCCGCATCAAGAGCAACCCATGCCTGATCGGAGAGCCAGGAATCGGCAAGACCGCCATCATCGAGGGAGTGGCCCAGCGCATCATCGATGATGATGTGCCAACCATCCTGCAGGGAGCCAAGCTGTTCAGCCTGGATCTGGCCGCCCTGACCGCCGGCGCCAAGTACAAGGGCGATTTCGAGGAGCGCTTCAAGGGCGTGCTGAAGGAGATCGAGGAGAGCAAGACCCTGATCGTGCTGTTCATCGATGAGATCCACATGCTGATGGGCAACGGCAAGGATGATGCCGCCAACATCCTGAAGCCAGCCCTGAGCCGCGGACAGCTGAAGGTCATCGGAGCCACCACCAACAACGAGTACCGCAGCATCGTGGAGAAGGATGGAGCCTTCGAGCGCCGCTTCCAGAAGATCGAGGTGGCCGAGCCAAGCGTGCGCCAGACCGTGGCCATCCTGCGCGGACTGCAGCCCAAGTACGAGATCCACCACGGCGTGCGCATCCTGGATAGCGCCCTGGTGACCGCCGCCCAGCTGGCCAAGCGCTACCTGCCATACCGCCGCCTGCCAGATAGCGCCCTGGATCTGGTGGATATCAGCTGCGCCGGAGTGGCCGTGGCCCGCGATAGCAAGCCAGAGGAGCTGGATAGCAAGGAGCGCCAGCTGCAGCTGATCCAGGTGGAGATCAAGGCCCTGGAGCGCGATGAGGATGCCGATAGCACCACCAAGGATCGCCTGAAGCTGGCCCGCCAGAAGGAGGCCAGCCTGCAGGAGGAGCTGGAGCCACTGCGCCAGCGCTACAACGAGGAGAAGCACGGCCACGAGGAGCTGACCCAGGCTAAGAAAAAGCTGGATGAGCTGGAGAACAAGGCCCTGGATGCCGAGCGCCGCTACGATACCGCCACCGCCGCCGATCTGCGCTACTTCGCCATCCCCGATATCAAGAAGCAGATCGAGAAGCTGGAGGATCAGGTGGCCGAGGAGGAGCGCCGCGCCGGCGCCAACAGCATGATCCAGAACGTGGTGGATAGCGATACCATCAGCGAGACCGCCGCCCGCCTGACCGGCATCCCCGTGAAGAAGCTGAGCGAGAGCGAGAACGAGAAGCTGATCCACATGGAGCGCGATCTGAGCAGCGAGGTGGTGGGCCAGATGGATGCCATCAAGGCCGTGAGCAACGCCGTGCGCCTGAGCCGCAGCGGACTGGCCAACCCACGCCAGCCAGCCAGCTTCCTGTTCCTGGGCCTGAGCGGCAGCGGCAAGACCGAGCTGGCCAAGAAGGTGGCCGGCTTCCTGTTCAACGATGAGGATATGATGATCCGCGTGGATTGCAGCGAGCTGAGCGAGAAGTACGCCGTGAGCAAGCTGCTGGGCACCACCGCCGGCTACGTGGGCTACGATGAGGGCGGCTTCCTGACCAACCAGCTGCAGTACAAGCCCTACAGCGTGCTGCTGTTCGATGAGGTGGAGAAGGCCCACCCCGATGTGCTGACCGTGATGCTGCAGATGCTGGATGATGGCCGCATCACCAGCGGCCAGGGCAAGACCATCGATTGCAGCAACTGCATCGTGATCATGACCAGCAACCTGGGCGCCGAGTTCATCAACAGCCAGCAGGGCAGCAAGATCCAGGAGAGCACCAAGAACCTGGTCATGGGCGCCGTGCGCCAGCACTTCCGCCCCGAGTTCCTGAACCGCATCAGCAGCATCGTGATCTTCAACAAGCTGAGCCGCAAGGCCATCCACAAGATCGTGGATATCCGCCTGAAGGAGATTGAGGAGCGCTTCGAGCAGAACGATAAGCACTACAAGCTGAACCTGACCCAGGAGGCCAAGGATTTCCTGGCCAAGTACGGCTACAGCGATGATATGGGCGCCCGCCCCCTGAACCGCCTGATCCAGAACGAGATCCTGAACAAGCTGGCCCTGCGCATCCTGAAGAACGAGATCAAGGATAAGGAGACCGTGAACGTGGTGCTGAAGAAGGGCAAGAGCCGCGATGAGAACGTGCCAGAGGAGGCCGAGGAGTGCCTGGAGGTGCTGCCAAACCACGAGGCCACCATCGGAGCCGATACCCTGGGCGATGATGATAACGAGGATAGCATGGAGATCGATGATGATCTGGATTAA

Multiple insertion lines were characterized for each transgene. To create the 1×gr-GAL4 driver line, the pGMR (glass multimer reporter) vector [96] was digested by XhoI/Acc651 to remove the insert containing five glass-binding sites. Complementary oligonucleotides (5′-TCGAACCCAGTGGAAACCCTTGAAATGCCTTTAACTCGAGACGG-3′ and 5′-GTACCCGTCTCGAGTTAAAGGCATTTCAAGGGTTTCCACTGGGT-3′), with a single copy of the 31 bp glass-binding site from the Rh1 proximal enhancer, were duplexed and ligated into the vector, producing p1×GR (1 copy of glass reporter). This plasmid was then modified to introduce the GAL4 coding sequence, excised from pGaTN [38] with HindIII, to create p1×gr-GAL4. MJD lines are from [41]. Experiments were performed at 25°C except for a select few conducted at 29°C as indicated. All results were confirmed with multiple UAS-Hsp104 insertion lines.

### Evaluation of eye degeneration

Eye images were obtained on day 7 of adulthood using a Leica Z-16 apo zoom microscope. To view internal retinal structure, heads were embedded in paraffin according to standard protocols, sectioned at 8 µm, and autofluorescence was viewed with a Leica fluorescence microscope. To quantify tissue loss, a standard area (3×15 rectangle; 7,000 µm^2^) (see also Fig. 9) was selected within the paraffin retinal section and the percentage of area covered by tissue was measured in ImageJ. Statistical analysis was performed using one-way ANOVA and unpaired t-test.

### Cryosections and immunohistochemistry

Heads were frozen in Tissue Freezing Medium (Electron Microscopy Sciences) and sectioned at 12 µm by cryotome, and the tissue sections were then fixed with 4% paraformaldehyde. Immunohistochemistry was performed according to standard procedures using primary antibodies anti-HA 5B1D10 (1∶100, Invitrogen 32-6700) or anti-myc 9E10 (1∶100, Santa Cruz sc-40) (both mouse) alongside either anti-Hsp104 (1∶100, Enzo Life Sciences ADI-SPA-1040) or anti-Hsp70 (1∶100, Enzo Life Sciences ADI-SPA-812) (both rabbit). Hsp70 staining was confirmed with human-specific anti-Hsp70 (1∶100, Santa Cruz sc-24) (mouse) alongside anti-HA Y11 (1∶100, Santa Cruz sc-805) or anti-myc A14 (1∶100, Santa Cruz sc-789) (both rabbit). Rabbit primary antibodies were preadsorbed at 1∶25 with fixed, dissected wild-type larvae. Secondary antibodies were Alexa Fluor 594 Goat-anti-Mouse IgG (1∶100, Life Technologies A-11032), Alexa Fluor 488 Goat-anti-Rabbit IgG (1∶100, Life Technologies A-11008), Alexa Fluor 594 Goat-anti-Rabbit IgG (1∶100, Life Technologies A-11037), and Alexa Fluor 488 Goat-anti-Mouse IgG (1∶100, Life Technologies A-11029). Sections were co-stained with Hoechst nuclear dye (1∶1000, Molecular Probes 33342) and viewed with a Leica fluorescence microscope. A 75 µm×75 µm square (5625 µm^2^) area was selected; particle analysis was performed with ImageJ and statistics performed with one-way ANOVA and unpaired t-test.

### Immunoblots and SDD-AGE

For Hsp104 expression level characterization, heads were ground with a pestle in NuPage LDS Sample Buffer, boiled for 3 min, run on NuPage 4–12% Bis-Tris gel, and semi-dry transferred onto nitrocellulose membrane. Antibodies used were anti-Hsp104 (1∶2000, Enzo Life Sciences ADI-SPA-1040) and anti-actin (1∶2000, Abcam ab8227) with secondary antibody Goat-anti-Rabbit-HRP (1∶5000, Chemicon AP307P). For MJD aggregation analysis through SDD-AGE (Semi-Denaturing Detergent Agarose Gel Electrophoresis) and accompanying Western immunoblots, heads were ground in lysis buffer (100 mM Tris pH 7.5, 50 mM NaCl, 10 mM β-Mercaptoethanol, and Roche complete mini EDTA-free protease inhibitor cocktail tablets) [48] and an aliquot was taken for evaluation of soluble material by Western immunoblot, as above. To the remaining sample, 4× Sample Buffer (2× TAE, 20% glycerol, 8% SDS, bromophenol blue) was added to final concentration 1×. The samples were run on a 1.5% agarose gel with 0.1% SDS in a running buffer of 1× TAE (40 mM Tris, 20 mM Acetic acid, 1 mM EDTA, pH 8.3) containing 0.1% SDS, and then transferred overnight onto nitrocellulose membrane using downward capillary transfer [48]. Antibodies used were anti-HA-conj-HRP 3F10 (1∶500, Roche 12013819001), anti-myc 9E10 (1∶500, Santa Cruz sc-40) followed by Goat-anti-Mouse-HRP (1∶2000, Jackson ImmunoResearch 115-035-146), and anti-tubulin-conj-HRP (1∶1000, Cell Signaling 11H10). All immunoblots were imaged using a FujiFilm LAS-3000 imaging system and quantification was performed in ImageJ and statistically analyzed by one-way ANOVA and unpaired t-test.

### Gene switch protocol

A 4.0 mg/ml stock solution of RU486 (Sigma M8046) was prepared in 100% ethanol, and then 50 µl (200 µg) was added to pre-prepared food vials containing ∼12 ml of food and gently shaken overnight [97]. For control conditions, 50 µl of 100% ethanol was added to vials. Adult flies were aged in food treated with either RU486 or ethanol for the time periods indicated.
